# Comparison Between Erector Spinae Plane Block versus Serratus Anterior Plane Block Regarding Analgesia and Stress Response After Modified Radical Mastectomy: Randomized Controlled Trial

**DOI:** 10.5812/aapm-142189

**Published:** 2024-03-26

**Authors:** Ahmed Abd Elmohsen Bedewy, Maged Salah Mohamed, Hesham Mohamed Sultan, Moataz Salah Khalil

**Affiliations:** 1 Anesthesia, Department of Surgical Intensive Care and Pain Medicine, Faculty of Medicine, Helwan University, Helwan, Egypt; 2 Anesthesia, Department of Surgical Intensive Care and Pain Medicine, Faculty of Medicine, Cairo University, Giza, Egypt

**Keywords:** Erector Spinae Plane Block, Serratus Anterior Plane Block, Modified Radical Mastectomy, Stress Response, Analgesia

## Abstract

**Background:**

Modified radical mastectomy (MRM) is the primary surgical treatment for breast cancer, yet it leads to significant postoperative pain.

**Objectives:**

This randomized controlled trial evaluates the effects of an erector spinae plane block (ESPB) versus a serratus anterior plane block (SAPB) on post-MRM pain management and stress response reduction.

**Methods:**

Sixty individuals scheduled for unilateral MRM under general anesthesia from October 2021 to October 2022 were divided into three groups. Group A comprised 20 patients who received ultrasound-guided ESPB (20 mL of 0.25% bupivacaine). Group B included 20 patients who received ultrasound-guided SAPB (20 mL of 0.25% bupivacaine). Group C was treated with intravenous morphine based on pain scores. Anesthesia was induced using 2 μg/kg of fentanyl and 2 - 3 mg/kg of propofol. The study compared the three groups regarding pain scores using a numerical rating scale, serum cortisol levels, total fentanyl, and morphine consumption, changes in mean arterial blood pressure (MAP) and heart rate (HR) during surgery, and the occurrence of postoperative complications.

**Results:**

Statistically significant reductions in pain scores were observed in group A compared to groups B and C. Moreover, group A exhibited a significant decrease in postoperative morphine consumption, serum cortisol levels 1 hour post-surgery (P = 0.021), MAP, and postoperative vomiting and nausea compared to group B. Furthermore, groups A and B showed statistically significant improvements in all parameters compared to group C.

**Conclusions:**

The study demonstrates that ESPB provides superior analgesic effects compared to SAPB in patients undergoing MRM, with reduced morphine use and lower postoperative cortisol levels. Both blocks offer more effective pain control than intravenous morphine alone.

## 1. Background

In over 100 countries, breast cancer (BC) is the most commonly diagnosed cancer and the leading cause of cancer death among women (1). Modified radical mastectomy (MRM) stands as the prevalent surgical treatment for BC. Inadequate management of postoperative pain adversely impacts the body's physiological and psychological functions. Effective management of acute pain suppresses the surgical stress response, diminishes the need for opioids and general anesthetics, and maintains the immune response (2).

Pain poses a significant challenge and represents one of the most debilitating symptoms for BC patients, negatively affecting their functional status and quality of life (QoL). The initial symptom in BC patients is often a painless lump (3). Managing pain in BC patients necessitates a comprehensive evaluation of the patient and a critical analysis of the pain. A precise assessment of the characteristics of pain is essential for the flawless management of BC pain. The intensity of pain, as reported by the patient, is regarded as the gold standard for routine pain assessment. Therefore, effective pain management relies on consistent screening for early detection of pain, accurate description of the pain's characteristics such as onset, duration (acute or chronic), intensity, location, severity, underlying pathophysiology, association with treatment, breakthrough pain, and more (4).

Regional anesthesia can reduce the stress response triggered by surgical trauma (5). The initial use of the erector spinae plane block (ESPB) was for treating individuals suffering from severe chronic thoracic neuropathic pain, as well as those undergoing video-assisted thoracoscopic surgeries. These patients exhibited a remarkable response to the block (6). Bonvicini et al. were the first to report a clinical case where ESPB was used to manage postoperative pain following breast surgeries, leading to a rapid recovery. Two years later, the use of ESPB in breast surgeries saw a significant increase. However, the effectiveness of ESPB remains a topic of debate (7).

The ultrasound-guided serratus anterior plane block (SAPB) targets the lateral cutaneous branches of the intercostal nerves by injecting local anesthetics into the fascial plane between the latissimus dorsi and serratus anterior muscles, reducing pain along the anterolateral chest wall (8). The adoption of SAPB for providing analgesia in mastectomy procedures has increased due to its safety and ease of application (9).

There is a scarcity of literature comparing ESPB and SAPB in MRM cases, with few studies existing that compare the blocks to a control group.

## 2. Objectives

This study aimed to compare the effects of ESPB and SAPB on managing post-MRM pain and reducing the stress response.

## 3. Methods

This was a randomized, controlled, single-blinded clinical trial conducted on 60 female patients at Helwan and Cairo University hospitals from October 2021 to October 2022. 

Inclusion criteria included 60 female individuals aged between 35 to 60 years, with an American Society of Anesthesiologists (ASA) physical status of classes I and II and a body mass index (BMI) of ≥ 20 kg/m^2^ and ≤ 35 kg/m^2^, all undergoing unilateral MRM under general anesthesia.

Exclusion criteria consisted of known sensitivity or contraindications to drugs used in the study (including local anesthetics and opioids), a history of psychological disorders and/or chronic pain syndrome, contraindications to regional anesthesia such as local sepsis, pre-existing peripheral neuropathies, coagulopathy, clinical skin infiltration by the tumor, severe respiratory conditions (such as severe obstructive pulmonary disease with a forced expiratory volume/forced vital capacity (FEV1/FVC) < 50%, or severe restrictive pulmonary disease with a total lung capacity (TLC) < 40%, adult respiratory distress syndrome), severe cardiac disorders (e.g., heart failure), advanced liver disease (with liver enzymes elevated more than three times the normal range), advanced kidney disease (with a creatinine clearance < 40 mL/min), and pregnancy.

### 3.1. Sample Method

Patients were randomly divided into 10 blocks, each consisting of 6 patients, with 2 patients assigned to group A, 2 to group B, and 2 to group C, before the blocks were sequenced. Patients were allocated to these blocks using the closed opaque envelope method. Group A included 20 patients who received ultrasound-guided ESPB. Group B comprised 20 patients who received ultrasound-guided SAPB. Group C consisted of 20 patients who were administered intravenous morphine alone. The individual assessing the outcomes was blinded to the group allocations. The drug interventions were prepared by a pharmacist not involved in the study, and the blocks were performed by the same anesthesiologist, who thereafter had no further involvement in the study.

### 3.2. Ethical Considerations

Following approval from the Medical Ethics Committee of Helwan University, with approval number (67-2021), signed informed consent was obtained from all participants prior to their inclusion in the study. The study protocol was registered in the Pan African Clinical Trial Registry (ID: PACTR202309543331995).

### 3.3. Study Procedure

In the pre-operative patient assessment, participants were instructed on using the Numeric Pain Rating Scale to communicate their pain levels, where a score of 0 signifies no pain and a score of 10 indicates the most severe pain imaginable. Researchers obtained informed consent from the participants. A 20-gauge IV cannula was inserted, and a 2-milliliter blood sample was drawn to measure the initial serum cortisol level. Prior to the surgical procedure, all individuals were premedicated with an intravenous dose of midazolam ranging from 0.01 to 0.02 mg/kg, administered 30 minutes before the operation.

### 3.4. General Anesthesia

During the surgery, monitoring equipment such as an electrocardiogram (EKG), pulse oximeter, non-invasive arterial blood pressure monitor, and capnography were used. Intravenous ringer infusion was started at a rate of 15 mL/kg/hour. After preoxygenation with 100% oxygen, anesthesia was induced with 2 μg/kg of fentanyl and 2 - 3 mg/kg of propofol. Endotracheal tube intubation was facilitated by administering atracurium at a dose of 0.5 mg/kg, with an additional 0.1 mg/kg given every 30 minutes. To reduce postoperative symptoms of vomiting and nausea, all participants received IV ondansetron at a dose of 4 mg and dexamethasone at a dose of 8 mg.

Anesthesia was maintained with isoflurane in a 50% oxygen/air mixture, achieving an expired isoflurane concentration of 1.2. Ventilation settings were adjusted to maintain an end-tidal CO_2_ level between approximately 30 - 40 mmHg. An intravenous fentanyl dose of 0.5 μg/kg was administered whenever there was an increase of over 20% in either the heart rate (HR) or mean arterial blood pressure (MAP) of the participant relative to their baseline values. The total amount of fentanyl administered was also recorded. Hemodynamic parameters, including MAP, HR, oxygen saturation, and end-tidal CO_2_, were monitored before the induction of anesthesia and at 15-minute intervals during the surgery. After the completion of skin closure, the isoflurane administration was stopped, and the reversal process began with an IV injection of neostigmine at a dose of 0.05 mg/kg, along with atropine at a dose of 0.02 mg/kg. Following successful extubation, participants were transferred to the post-anesthetic care unit (PACU).

### 3.5. Group A: Erector Spinae Plane Block Technique

**Figure 1.**
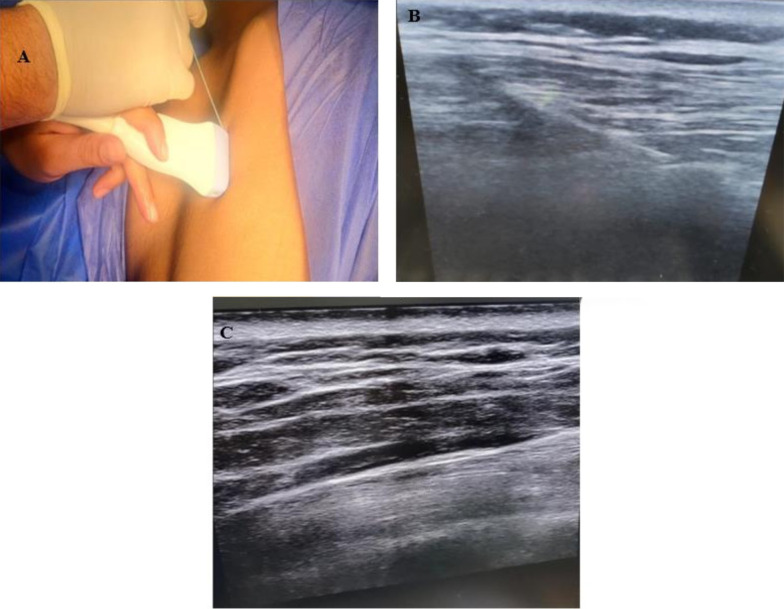
A, Erector spine block technique; B, after injection and withdrawal of the needle; C, with a needle in place

After anesthesia induction and 15 minutes before the skin incision, the block was performed with strict aseptic precautions. The procedure was executed with the patient's arm abducted and positioned laterally at the T5 level. To identify the tip of the T5 transverse process, an ultrasound probe was placed longitudinally on the posterior aspect, 3 cm away from the spine. This placement revealed distinctive flat, squared-off acoustic shadows, with the pleura faintly visible. Should the transducer be positioned too laterally, ribs would appear as spherical acoustic shadows, accompanied by a clearly visible hyperechoic pleural line. Following this, a 21-gauge 80 mm echogenic needle was inserted from cranial to caudal, aligned within the ultrasound beam. A volume of 20 milliliters of 0.25% bupivacaine solution was injected after ensuring there was no intravascular entry through aspiration. This resulted in a visible separation between the erector spinae muscle and the transverse processes (Figure 1). 

### 3.6. Group B: Serratus Anterior Plane Block Technique

**Figure 2.**
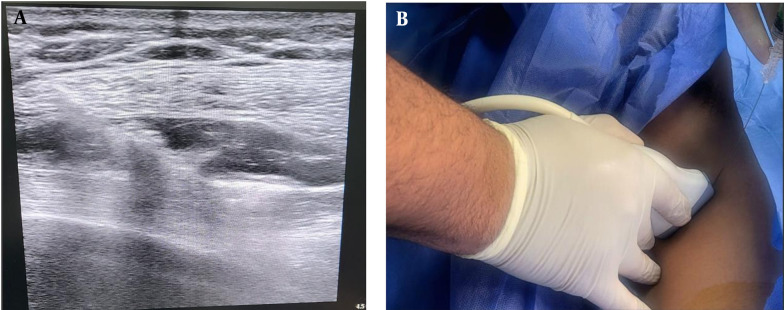
Serratus anterior plane block technique

After anesthesia was administered, the patient was positioned in a supine orientation, with the arm moved away from the surgical site 15 minutes before the operation began. Starting from the lower side, ribs were identified along the mid-axillary line up to the fifth rib. When the linear ultrasound probe was placed horizontally, three muscles were visualized: The superiorly located teres major, the superficial and posterior latissimus dorsi, and the deep and inferior serratus muscles. The thoracodorsal artery, located slightly posteriorly, served as a landmark for identifying the plane superficial to the serratus muscle. The needle was inserted in-plane relative to the ultrasound probe, moving from superior to inferior. Subsequently, a total of 20 mL of 0.25% bupivacaine was injected between the latissimus dorsi and serratus anterior muscles, with aspiration performed first to prevent intravascular injection. Both blocks utilized MINDRAY ultrasound equipment with a linear transducer set to 6 - 13 MHz, optimized for small parts, and a depth setting of 1 - 4 cm (Figure 2). 

Group C included patients who did not receive any blocks.

### 3.7. Postoperative Assessment

After surgery, participants from all three groups were transferred to the PACU. Upon arrival at the PACU, patients were immediately assessed for pain intensity using the Numerical Pain Scale, and a postoperative X-ray was conducted after PACU discharge to rule out pneumothorax (10). Further assessments occurred at 2, 4, 6, 12, and 24 hours postoperatively. These evaluations included monitoring hemodynamic parameters and determining pain severity with the Numerical Rating Scale (NRS). Intravenous morphine at a dosage of 0.05 mg/kg/dose was administered when the NRS score reached or exceeded 4. The total morphine consumption over a 24-hour period was recorded for these individuals. Blood samples were collected 1 hour postoperatively to measure serum cortisol levels. The samples, stored in serum tubes, were centrifuged and then kept at -20°C until analysis by enzyme-linked immunosorbent assay (ELISA). Adverse effects such as vascular damage, hypotension, pneumothorax, or local infection were noted, along with complications like nausea, vomiting, postoperative respiratory depression (11), and the Ramsay sedation score (12).

### 3.8. Sample Size

To perform a two-sided two-sample *t*-test, our objective is to achieve a statistical power of 80% to detect a difference of 6.0 between the null hypothesis, which posits that the means of the two groups are equal at 16.7, and the alternative hypothesis, which proposes that the mean of group 2 is 10.7. The estimated standard deviations for the two groups are 7.2 and 3.1, respectively. The significance level (alpha) is set at 0.05. To accommodate potential participant dropouts and address attrition in prospective research, we increased the sample size by 20%.

### 3.9. Statistical Analysis

Data were collected by a researcher who was not involved in administering the blocks. All data were compiled and analyzed statistically using SPSS version 26.0 for Windows (SPSS Inc., Chicago, IL, USA). Quantitative data were expressed as means ± SD and median (range), while qualitative data were presented as absolute frequencies (number) and relative frequencies (percentage) and analyzed using the ANOVA (F) test with a post hoc Tukey test. Non-parametric quantitative data were expressed as the median and interquartile range (IQR) and analyzed using the Kruskal-Wallis test with the Mann-Whitney test for comparisons between groups. Qualitative variables were presented as frequency and percentage (%) and analyzed using the chi-square test. A two-tailed P-value of less than 0.05 was considered statistically significant.

## 4. Results

**Figure 3.**
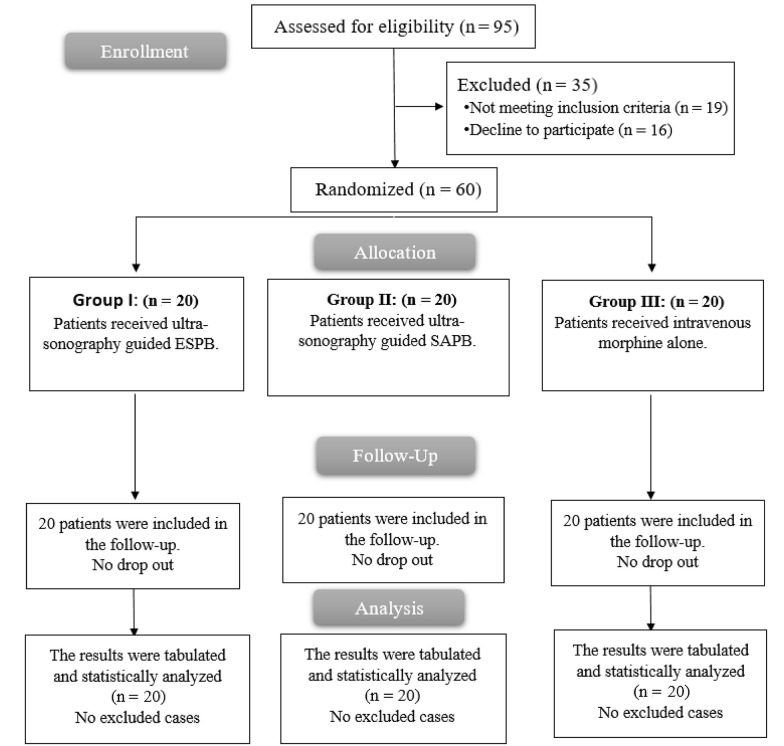
The flow chart of the studied groups

In this study, 95 patients were assessed for eligibility; 19 patients did not meet the criteria, and 16 patients declined to participate. The remaining 60 patients were randomly divided into three equal groups (20 patients in each group). All allocated patients were followed up and analyzed statistically (Figure 3). 

There was no statistically significant difference among the groups in terms of age, weight, and surgery duration, ensuring the groups were comparable (Table 1). 

**Table 1.** Patients’ Basic Data in the Studied Groups ^a^

Variables	Group A (n = 20)	Group B (n = 20)	Group C (Control) (n = 20)	P-Value
**Age (y)**				0.794
35 - 59	47 ± 7.54			
37 - 55		45.65 ± 5.40		
38 - 56			46.60 ± 6.18	
**Weight (kg)**				0.656
65 - 98	80.60 ± 8.68			
66 - 98		80.95 ± 8.83		
66 - 99			83.05 ± 9.75	
**Duration of surgery (min)**				0.444
70 - 95	83.50 ± 7.16			
65 - 92		80.65 ± 7.26		
66 - 99			83.20 ± 8.64	

^a^ Values are expressed as mean ± SD.

**Figure 4.**
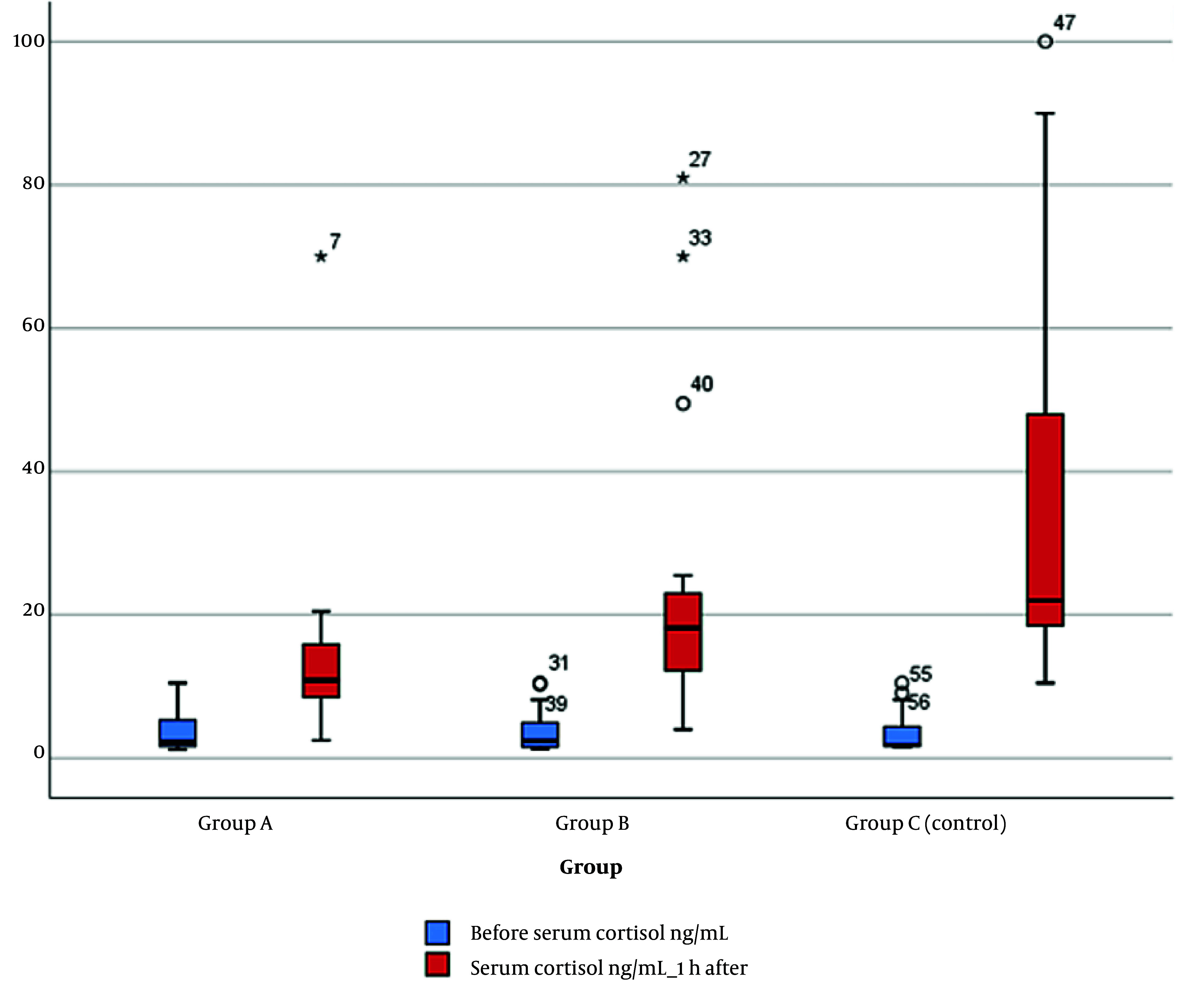
Box blot chart serum cortisol level before and 1 hour after surgery in the studied groups

The serum cortisol level 1 hour after surgery significantly decreased in group A compared to group B (P = 0.021) and compared to group C (P = 0.001). A significant reduction was also observed between groups B and C (P = 0.026), while there were no significant differences among the groups before the operation (Figure 4). 

Group A exhibited a significant reduction in total morphine consumption compared to groups B (P = 0.044) and C (P = 0.001). Group B also consumed less morphine than group C (P = 0.001). There was no significant difference between groups A and B regarding fentanyl consumption; however, a significant difference was observed between groups A and C, as well as between groups B and C. Furthermore, significant differences were noted among the groups in terms of changes in MAP at various intervals (P < 0.05), with group A showing the lowest MAP at several intervals (PACU, 2 hours, 4 hours, and 6 hours). No significant difference was found between groups A and B at 4, 12, and 24 hours, yet significant differences were noted between groups A and C and between groups B and C at most intervals (Table 2). 

**Table 2.** Intra-Operative Fentanyl Consumption and Total Morphine Consumption in the Studied Groups

Variables	Group A ^a^ (n = 20)	Group B ^a^ (n = 20)	Group C ^a^ (Control) (n = 20)	Tests		Post Hoc (Mann-Whitney Test) ^c^
				Test	P-Value ^c^	
**Intraoperative fentanyl consumption (mcg)**	43 (35.5 - 47.5)	41.25 (38.13 - 62)	100 (88.5 - 128)	F ^b^ = 34.636	< 0.001	P1 = 0.924; P2 < 0.001; P3 < 0.001
**Total morphine consumption (mg)**	4.18 (3.29 - 6.38)	8.5 (3.4 - 11.25)	16.25 (13.4 - 18)	F ^b^ = 36.454	< 0.001	P1 = 0.044; P2 < 0.001; P3 < 0.001

^a^ Values are expressed as median (IQR).

^b^ F: ANOVA.

^c^ P-values < 0.05 are significant.

There was a significant difference among the groups regarding the incidence of nausea and vomiting (P < 0.05). Approximately 10% of patients in group B experienced nausea and vomiting and other adverse effects such as vascular damage, hypotension, pneumothorax, or local infection, while no patients in group A reported any postoperative complications. Group C had the highest incidence of vomiting and nausea, but there was no significant difference between the groups regarding postoperative respiratory depression (Table 2). 

There was a statistically significant difference between the studied groups regarding pain scores at various intervals (P < 0.05), with group A displaying the lowest pain scores at most intervals. Significant differences were observed between group A and group C, as well as between group B and group C, at all intervals (Table 3, Figure 4). 

**Table 3.** Pain Score at Different Intervals Between the Studied Groups

Variables	Group A ^a^ (n = 20)	Group B ^a^ (n = 20)	Group C ^a^ (Control) (n = 20)	Tests		Post Hoc (Mann-Whitney Test) ^b, c^
				Kruskal-Wallis Test	P-Value ^b^	
**PACU**	1 (1 - 2)	2.5 (1 - 3)	5.5 (4 - 7)	30.5	< 0.001	P1 = 0.011; P2 < 0.001; P3 < 0.001
**2 hours**	2 (1 - 3)	2 (1.3 - 4)	4 (3 - 5)	12.37	0.002	P1 = 0.435; P2 = 0.001; P3 = 0.011
**4 hours**	2 (1 - 3)	2.5 (2 - 4)	3.5 (2.25 - 4.75)	11.14	0.004	P1 = 0.211; P2 = 0.001; P3 = 0.035
**6 hours**	2 (1.25 - 3)	2 (2 - 3)	4.5 (3.25 - 5.75)	21.89	< 0.001	P1 = 0.244; P2 < 0.001; P3 = 0.001
**12 hours**	4 (3.25 - 5.75)	3 (2 - 3.8)	4 (3 - 4)	8.95	0.011	P1 = 0.006; P2 = 0.044; P3 = 0.030
**24 hours**	3 (2 - 3)	2.5 (1.25 - 3)	4.5 (2.25 - 5.75)	13.56	0.001	P1 = 0.732; P2 = 0.002; P3 = 0.002

^a^ Values are expressed as median (IQR).

^b^ P-values < 0.05 are significant.

^c^ P1 = group A vs. group B; P2 = group A vs. group C; P3 = group B vs. group C.

## 5. Discussion

This study documented pain scores using the NRS at 0, 2, 4, 6, 12, and 24 hours post-operation. A statistically significant difference was found among the groups concerning pain scores at different intervals (P < 0.05).

In research conducted by Jiang et al., it was found that NRS scores in the ESPB group were significantly lower than those in the SAB group at various time points (0.5, 1, 3, 6, 12, 18, and 24 hours post-operation) when the patients were active (P < 0.05 for all comparisons) (13).

Sharma et al. observed decreased pain scores in the block group compared to the control group at all intervals (at 0 hours P = 0.017; at 0.5 hours P = 0.001; at 1 hour P = 0.01, at 2 hours P = 0.002, at 4 hours P = 0.012, at 6 hours P < 0.001, at 12 hours P = 0.009, and at 24 hours P = 0.006), except at the 8-hour interval (P = 0.137) (14).

Yao et al.'s results indicated that the serratus plane block group experienced lower pain scores at rest throughout the first 24-hour postoperative period compared to the control group (P < 0.001) (9).

We also observed a statistically significant difference among the groups concerning postoperative serum cortisol levels, indicating a reduction in stress hormone levels in group A compared to groups B and C, as well as between groups B and C. Su et al. noted a decrease in blood cortisol levels and other stress hormones within the first 24 hours after surgery (P < 0.001) (15). Yamamoto et al. found no significant difference in postoperative serum cortisol levels between the SAPB group and the thoracic epidural group, suggesting that the SAPB is comparable to thoracic epidural anesthesia in reducing stress hormone levels after surgery (16).

A significant decrease in postoperative morphine consumption was observed among groups A, B, and C (P < 0.05), which could also be attributed to the analgesic effects of these blocks. Jiang et al. reported a significant reduction in 24-hour postoperative opioid consumption in the ESP group compared to the SAB group (P < 0.05) (13). Seelam et al. observed a highly significant reduction in postoperative morphine usage between the ESP group and the control group (P < 0.001) (17). Yao et al. noted a significant difference in 24-hour morphine intake between the SAB group and the control group (P < 0.001) (9).

There was no statistically significant difference between groups A and B regarding intraoperative fentanyl use (P = 0.924). Additionally, we found a highly significant decrease in the intraoperative need for fentanyl among groups A and C (P < 0.001) and between groups B and C (P < 0.001). Jiang et al. reported no significant difference in the need for intraoperative opioids between the ESP and SAB groups (P = 0.945) (13).

According to the findings, the ESPB offered marginally superior analgesia in breast surgeries compared to the SAPB, and it more effectively reduced the postoperative stress response than the SAB block. This led to a reduced need for morphine in the postoperative period, which in turn resulted in a lower incidence of postoperative nausea and vomiting (PONV). The observed results could be attributed to the fact that, unlike the SAP block, the ESP block affects both the dorsal and ventral rami of the thoracic spinal nerves. Additionally, this block induces a level of sympathetic suppression, in contrast to the SAP block, which primarily targets the intercostal nerve branches. However, cadaveric and MRI studies provide inconsistent data on whether local anesthesia consistently reaches the paravertebral area with ESP blocks. Both groups exhibited a significantly lower stress response and better analgesia compared to the control group, which could be due to the effect of these blocks in mitigating the stress response and preventing the cascade of events that typically follow, resulting in a reduced need for opioids and fewer complications (18).

While the study had several strengths, such as comparing two different blocks (ESPB and SAPB) in patients undergoing mastectomy versus a control group without blocks—thus providing evidence of the optimal nerve block and establishing a causative relationship between the outcomes—it also faced limitations, including being a single-center study with a small sample size and a short follow-up period. Additionally, the study lacked a comprehensive identification of adverse effects associated with both blocks. Therefore, further studies with larger sample sizes are recommended, as well as exploring different local anesthetics (LAs) with varying blocks, volumes, and concentrations.

### 5.1. Conclusions 

In conclusion, the ESPB demonstrates a slightly higher analgesic effect than the SAPB in breast surgeries, and both blocks yielded better outcomes than morphine alone. However, larger studies are necessary to confirm these findings of improved analgesia and reduced stress response.

## Data Availability

The dataset presented in the study is available on request from the corresponding author during submission or after publication.

## Clinical Trial Registration Code:

PACTR202309543331995.
